# First Molecular Epidemiology Study of *Mycobacterium tuberculosis* in Kiribati

**DOI:** 10.1371/journal.pone.0055423

**Published:** 2013-01-31

**Authors:** Eman Aleksic, Matthias Merker, Helen Cox, Bereka Reiher, Zamberi Sekawi, Anna C. Hearps, Claire E. Ryan, Adele V. Lee, Regis Goursaud, Clement Malau, Janet O'Connor, Catherine L. Cherry, Stefan Niemann, Suzanne M. Crowe

**Affiliations:** 1 Centre for Virology, Burnet Institute, Melbourne, Australia; 2 Molecular Mycobacteriology, National Reference Center for Mycobacteria, Forschungszentrum, Borstel, Germany; 3 Department of Medicine, Monash University, Victoria, Australia; 4 TB Clinic, Tungaru Central Hospital, Tarawa, Kiribati; 5 Faculty of Medicine and Health Sciences, University Putra Malaysia, Serdang, Selangor, Malaysia; 6 Department of Microbiology, Pasteur Institute, Noumea, New Caledonia; 7 Nossal Institute of Health, The University of Melbourne, Victoria, Australia; 8 Secretariat of the Pacific Community, Noumea, New Caledonia; 9 Department of Infectious Diseases, The Alfred Hospital, Melbourne, Australia; St. Petersburg Pasteur Institute, Russian Federation

## Abstract

Tuberculosis incidence rates in Kiribati are among the highest in the Western Pacific Region, however the genetic diversity of circulating *Mycobacterium tuberculosis* complex strains (MTBC) and transmission dynamics are unknown. Here, we analysed MTBC strains isolated from culture positive pulmonary tuberculosis (TB) cases from the main TB referral centre between November 2007 and October 2009. Strain genotyping (IS*6110* typing, spoligotyping, 24-loci MIRU-VNTR and SNP typing) was performed and demographic information collected. Among 73 MTBC strains analysed, we identified seven phylogenetic lineages, dominated by Beijing strains (49%). Beijing strains were further differentiated in two main branches, Beijing-A (n = 8) and -B (n = 28), that show distinct genotyping patterns and are characterized by specific deletion profiles (Beijing A: only RD105, RD207 deleted; Beijing B: RD150 and RD181 additionally deleted). Many Kiribati strains (59% based on IS*6110* typing of all strains) occurred in clusters, suggesting ongoing local transmission. Beijing-B strains and over-crowded living conditions were associated with strain clustering (likely recent transmission), however little evidence of anti-tuberculous drug resistance was observed. We suggest enhanced case finding amongst close contacts and continued supervised treatment of all identified cases using standard first-line drugs to reduce TB burden in Kiribati. Beijing strains can be subdivided in different principle branches that might be associated with differential spreading patterns in the population.

## Introduction

Tuberculosis is a major global health challenge with an estimated 9 million new active cases and 1.45 million deaths annually, with control efforts threatened by the emergence of drug resistance [1], [2]. The Western Pacific Region (WPR) accounts for 21% of the global TB burden [2], [3]. Kiribati is a small, widely scattered Western Pacific nation consisting of 32 coral atolls and one island covering an area of 812 square km, with a population of 103 466 [4] and a high incidence of TB (estimated 370/100 000 population [1]). Detailed data on factors contributing to the relatively high incidence of TB in Kiribati and other smaller Pacific Island countries and territories are lacking, hampering the development of improved public health measures for TB control in the area.

The Secretariat of the Pacific Community, New Caledonia, and World Health Organization play an essential role in collating basic regional surveillance data, supporting the local National TB program (NTP) [5]–[7], and strengthening the limited infrastructure in Kiribati. The Kiribati NTP is based in the capital, South Tarawa, and works with 21 surrounding island councils to provide TB services [8], with patients referred to one of two sites for diagnosis and treatment (South Tarawa atoll or Kiritimati island). The Kiribati NTP provides sputum smear testing and directly observed short course TB therapy (DOTS) at no cost to patients and includes the availability of infant Bacillus Calmette–Guérin (O'Connor J, personal communication). The estimated TB incidence rate in 1995 was 505/100 000. Introduction of DOTS was associated with a reduction to 370/100 000 (2010) [1], assisted by innovative approaches to deliver TB medication to patients by motorcycle and the building of a ‘maneaba’ (traditional meeting place for TB patients) designed to increase natural air flow [8]. Little is known about the success of treatment or of drug susceptibility of *Mycobacterium tuberculosis* complex (MTBC) strains in Kiribati. There has already been at least one case of multi-drug resistant pulmonary TB in Kiribati in 2005 (the patient died within a year of diagnosis and no family members were subsequently diagnosed with TB; O'Connor J, personal communication). Only two laboratories in Kiribati are capable of performing acid fast bacilli (AFB) smears and neither MTBC culture nor drug susceptibility testing (DST) are routinely available in-country [1].

Molecular epidemiology is a useful tool for analysing MTBC strain diversity and transmission patterns in both low and high incidence settings [9], [10]. The specific aims of this study were to identify and evaluate the diversity of circulating MTBC strains, investigate parameters associated with TB transmission and to evaluate the level of drug resistance in Kiribati.

## Materials and Methods

### Study population and sputum handling

All patients with clinically suspected pulmonary TB who presented to the main TB centre in Kiribati at Tungaru Central Hospital, South Tarawa between November 2007 and October 2009 were eligible for this study, regardless of sputum smear (SS) result. Diagnosis of SS positive TB was confirmed by microscopic examination of three consecutive Ziehl-Neelsen stained sputum smears. Patients answered a brief medical and demographic questionnaire.

Sputum from each study patient was refrigerated and transported to Réseau International des Institut Pasteur (Noumea, New Caledonia) for culture of MTBC strains. Samples were decontaminated by the modified dodecyl (*lauryl*) sulfate-NaOH method [11], cultured on Löwenstein-Jensen medium (BioMérieux, France) and incubated (37°C) with growth read on days 28, 56 and 84 before final negative culture status. Identification of primary isolates was performed using the AccuProbe® Mycobacterium Tuberculosis Complex kit (Gen-Probe, San Diego, California, U.S.A.). DST was completed in the Pasteur-CERBA laboratory, Paris, using the Bactec® MGIT® 960 SIRE Kit (BD Diagnostic System, France) using a modified MiddleBrook 7H9 medium with two critical concentrations (1 µg/ml and 4 µg/ml) of isoniazid, rifampicin, streptomycin (STR) and ethambutol. Genomic DNA was extracted using an InstaGene Matrix® (BioRad, California, U.S.A.).

### Ethics Statement

Ethical clearance was obtained from The Alfred hospital human research ethics committee, Melbourne, Australia (study 22/06) and from the Ministry of Health, Kiribati, with recruited participants giving written consent. Written informed consent for use of clinical data (including access to HIV test results) was obtained from patients or on the behalf of minors/children participants from their next of kin, caretakers or guardians.

### Genotyping

Extracted DNA was sent frozen to the Burnet Institute (Melbourne, Australia) and the Molecular Mycobacteriology laboratory (Borstel, Germany) for spoligotyping [12], 24-loci mycobacterium interspersed repetitive unit-variable number of tandem repeat (MIRU-VNTR) typing [13] and IS*6110*-Restriction Fragment Length Polymorphism (RFLP) [14]. Customized kits were used for MIRU-VNTR typing (Genoscreen, Lilli, France).

Briefly, MIRU-VNTR analyses were performed using multiplex PCRs, the Rox-labeled MapMarker 1000 size and MapMarker 1,500 size standards (only mix 5; Bio-Ventures, Inc., Murfreesboro, VT), and ABI 3130 XL sequencer with 16 capillaries (Applied Biosystems, Foster City, CA). Sizing of PCR fragments and assignment of VNTR alleles used customized Genotype software packages (Applied Biosystems). External quality control panel produced by the European Centre for Disease Prevention and Control & Dutch National Institute for Public Health and the Environment groups (2010) for 24-loci MIRU-VNTR typing was performed by the Borstel laboratory obtained typing results matching 100% with the reference data (30 out of 30 strains tested) with 100% Intra-Laboratory reproducibility.

### Data analysis

The IS*6110* fingerprints, spoligotyping patterns, and MIRU-VNTR typing results of MTBC isolates were analysed by the Bionumerics program (Windows XP, Version 6.5; Applied Maths, Kortrijk, Belgium) as described previously [14]. The IS*6110* patterns were digitised and analysed for similarity using the Dice coefficient and a band-matching tolerance of 1.2%. MIRU-VNTR data were analysed using the categorical coefficient. For both methods, similarity trees were calculated using the UPGMA (unweighted pair group method with arithmetic averages) method as instructed by the manufacturer. Minimum Spanning Tree (MST) analysis based on MIRU-typing data was performed using the categorical coefficient. The priority rule was to link types first that had highest number of single-locus variants.

Clusters were defined as groups of MTBC strains showing either identical RFLP patterns (the same number of IS*6110* bands at identical positions) and spoligotyping patterns or identical MIRU-VNTR and spoligotyping patterns. Identification of MTBC phylogenetic lineage and dendrogram was created utilising Distance Measure: MIRU-VNTR [24]: Dc [15] using the MIRU-VNTR*plus* database [16].

### Single Nucleotide Polymorphism (SNP) Analysis

Phylogenic strain classification based on MIRU-VNTR and spoligotyping typing was confirmed by using lineage specific SNPs in Rv0557, Rv2629, and Rv0129c [17]. These SNPs allow highly specific and sensitive classification of clinical isolates in the following lineages: Euro-American super lineage (not Rv0557_321 t>c), *M. africanum* West African 1 (Rv0557_221 c>t), East African Indian (EAI, Rv0557_810 c>t), *M. caprae* (Rv0557_1050 g>a), Haarlem (Rv0557_455 g>c), S-type (Rv0557_457 c>g), Cameroon (Rv0557_532 c>g), Beijing (Rv2629_191 a>c), and Latin American Mediterranean (LAM, Rv309 g>a) [17]. To determine SNP, the respective genes were PCR amplified and sequenced. Analysis of sequence data and SNP detection was performed using SeqScape v2.6 (Applied Biosystems) and compared to the H37Rv reference genome for strain classification. Beijing strain classification was confirmed by presence or absence of RD105 using protocols available at the MIRU-VNTR*plus* webpage [16].

### Analysis of Beijing specific regions of difference (RD105, RD207, RD150 and RD181)

To further differentiate strains belonging to the Beijing lineage (identified by the characteristic spoligotype, showing absence of spacer 1–34) we analysed all Beijing strains by PCR to determine the presence or absence of specific regions of difference (RD). For further differentiation Beijing strains were classified Beijing-A and –B (Beijing A: only RD105, RD207 deleted; Beijing B: RD150 and RD181 additionally deleted). Genomic regions were identified as previously described [18]. A modified PCR amplification was performed using the QIAGEN HotStar Taq kit (Qiagen, Hilden, Germany) using 4–8 ng DNA, with final concentrations of 5% DMSO (Merck, Darmstadt, Germany), 2.25 mM MgCl_2_, 200 µM of each dNTP, 0.5 µM of each primer and 0.625units HotStar Taq DNA polymerase (Qiagen, Hilden, Germany) in a 25 µL reaction mixture. Optimised PCR cycle conditions were 95°C for 15 min, 30 cycles of 94°C for 30 s, 65°C for 30 s, 72°C for 3 min and a final extension of 72°C for 15 min. The following primers were used: RD 105Fwd (GCTTGCCGGGTGGGGTGGAGGTC), RD 105int (CGGCTGGCGGGCAATGTTT), RD 105Rev (CGCCGCGCCGTGGTGAACA), RD 207Fwd (CCTGCGGCGGCGGATGAAACT), RD 207int (CGCCGCGGATCAGATTGCTCACT), RD 207Rev (TGCCCCGCGGACACCCTCTACTCT), RD 181Fwd (TCGGCGGCCTCACGGATGGATT), RD 181int (CTTGGTGGTGGCTGGCCTGGTTCG), RD 181Rev (CGGGCGGCTGCGGGAACCTT), RD 150Fwd (GCTTGCCGGGTGGGGTGGAGGTC), RD 150int (CGGCTGGCGGGCAATGTTT), RD 150Rev (CGCCGCGCCGTGGTGAACA).

### Additional statistical analyses

Patient clinical and demographic details, associations with sputum culture results and associations between patient/sputum details and TB strain and clustering were analysed using Stata version 10.1 (Statacorp, College Station, Texas, USA). Descriptive statistics are presented as number (percentage) or median (range). Univariate comparisons were performed using Chi square tests, Wilcoxon rank-sum test or Fisher's exact test, as appropriate. Multivariate analyses to determine factors independently associated with positive culture, specific TB strains or clustering were performed using logistic regression modelling, including all factors with p<0.1 on univariate analyses and a stepwise removal procedure.

## Results

### Study population and TB culture results

One hundred and sixty-three patients presented to Tungaru Central Hospital with suspected pulmonary TB during the study period and consented to participate in this research (representing 37% of the 445 suspected pulmonary TB cases seen nationally in the study period [Source: Kiribati National TB Register]). The demographic characteristics of the study participants are shown in Table 1. Results of HIV testing were recorded for only nine patients (5.5%). No patient was found to be HIV infected. The majority (91.4%) of patients' sputum samples were smear positive for AFB, however only 74 (45.4%) were culture positive for MTBC. Factors associated with culture positivity were the number of AFB seen on microscopy (samples with more AFB seen were more likely to be culture positive) and the time taken for the sample to reach the culture facility in Noumea (longer delays were associated with negative culture; Table 2).

**Table 1:** Demographic details of study participants.

	Study participants (n = 163)	TB cases NOT in clusters (n = 30)	TB cases in clusters (n = 43)	p value (clustered vs non clustered cases)
**Born in Kiribati**	163 (100%)	30 (100%)	43 (100%)	1.0^1^
**Ever lived overseas**	0 (0%)	0 (0%)	0 (0%)	1.0^1^
**Age in years [median(range)]**	29 (9–77)	35 (14–75)	32 (10–75)	0.72^2^
**Female gender**	73 (44.8%)	18 (60%)	12 (40%)	0.46^1^
**Live on South Tarawa (most populous area served by the study hospital)**	117 (71.8%)	21 (70%)	32 (74.4%)	0.68^1^
**Occupation**				
***Paid employment***	30 (18.4%)	4 (13.3%)	9 (20.9%)	0.47^1^
***Homemaker***	73 (44.8%)	18 (60%)	19 (44.2%)	
***Student***	42 (25.8%)	5 (16.7%)	10 (23.3%)	
***Fisherman***	12 (7.4%)	2 (6.7%)	1 (2.3%)	
***Other***	6 (3.7%)	1 (3.3%)	4 (9.3%)	
**Number of people in the home [median(range)]**	8 (1–60)	8.5 (2–20)	9 (3–60)	0.24^2^
**People per room at home [median(range)]**	4 (0.5–17)	3.1 (0.8–14)	5 (1.6–15)	0.1^2^
**Prior TB diagnosis?**	6 (3.7%)	1 (3.3%)	2 (4.7%)	0.6^3^
**Documented HIV test in medical file**	9 (5.5%)^4^	2 (6.6%)	4 (9.4%)	0.6^3^

1 Chi square test.

2 Wilcoxon Rank Sum test.

3 Fisher's Exact test.

4 All available HIV serology was negative. In Kiribati, HIV testing is generally performed using the Determine HIV1/2 kit as an initial screening assay (Alere, Queensland, Australia).

**Table 2:** Factors associated with sputum sample being culture positive.

	Culture POS (n = 74)	Culture NEG (n = 89)	p
**Microscopy result**			
Negative	0 (0%)	14 (100%)	0.003^1^
Scanty	8 (40%)	12 (60%)	
**+**	15 (43%)	20 (57%)	
**++**	19 (49%)	20 (51%)	
**+++**	32 (58%)	23 (42%)	
**Time (days) to Noumea [median (range)]**	35.5 (3–165)	58 (7–293)	0.003^2^

1 Chi square test.

2 Wilcoxon Rank Sum test.

### Drug susceptibility of TB strains

Seventy-three of 74 (99%) MTBC strains successfully underwent susceptibility testing to four first line anti-tuberculous agents. No multi-drug resistant strains were identified. Seventy one strains were fully susceptible, one was STR resistant and two demonstrated intermediate susceptibility to STR (MIC 1–4 µg/ml). The three patients from whom strains with reduced STR susceptibility were isolated all had negative HIV serology recorded, did not report previous TB treatment and lived on South Tarawa.

### *M. tuberculosis* population structure and cluster analysis

High molecular weight chromosomal DNA was isolated from 73 of 74 (99%) MTBC strains and successfully underwent 24-loci MIRU typing, spoligotyping, IS*6110* DNA fingerprint analysis and sequencing of the target genes for SNP based phylogenetic classification. Initial population structure identification of Kiribati MTBC strain phylogenetic lineages were based on 24-loci MIRU typing and spoligotyping data. Genotyping data were compared with strains in the MTBC reference collection of the MIRU-VNTR*plus* webpage [16].

**Figure 1:**
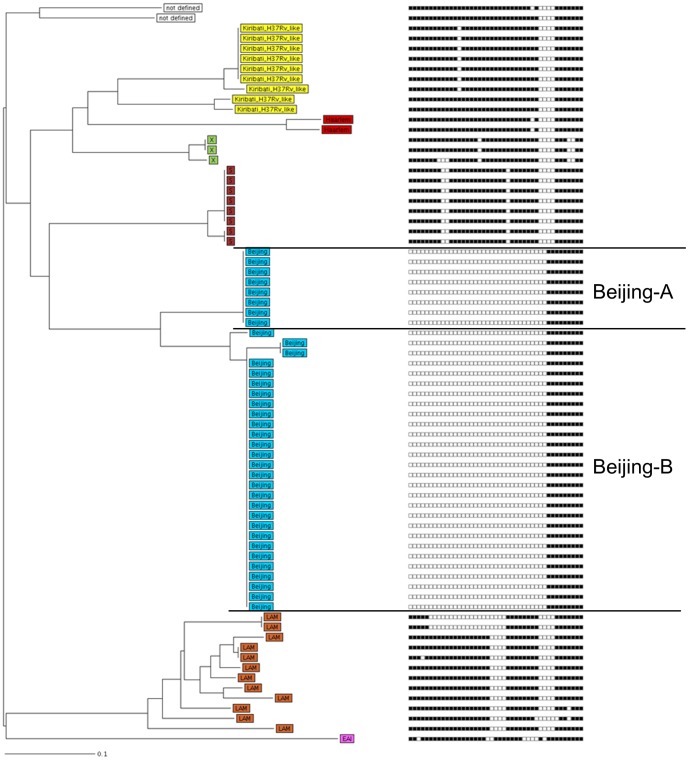
Neighbour joining tree combining 24 loci MIRU-VNTR and spoligotyping data. Neighbour joining tree (left panel) based on the copy numbers of 24 loci MIRU-VNTR (right panel, spoligotyping results whereby a repeat region's presence or absence is indicated by a black box or white box respectively) of the 73 strains investigated. The tree was calculated using the MIRU-VNTR*plus* server (Distance Measure: Dc: Cavalli-Sforza and Edwards, 1967: Dc [15]). Seven lineages were identified including 71 out of the 73 strains (97%), while two strains could not be allocated to a strain family. Classification of the strains into the different phylogenetic lineages is visualized by color coding, key on Figure 2.

**Figure 2:**
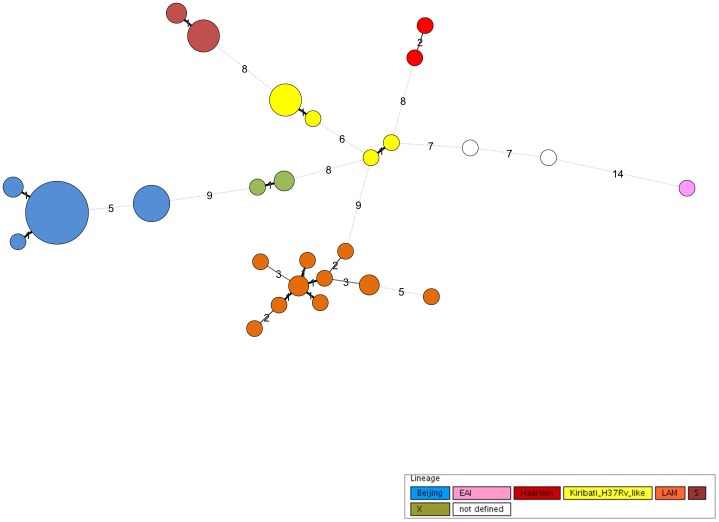
Minimum Spanning Tree of 24 loci MIRU-VNTR data. Minimum Spanning Tree, based on the 24 loci MIRU-VNTR typing data of 73 analysed Kiribati strains. The size of each circle is proportional with the number of MIRU-VNTR types belonging to a particular complex. Classification of the strains into the different phylogenetic lineages is visualized by color coding.

Each 24-MIRU-VNTR combination was also assigned a unique MTBC 15-9 number (Table S1) using the nomenclature server of MIRU-VNTR*plus* [16]. These analyses classified 62 of the Kiribati MTBC strains into the following lineages: Beijing (n = 36), LAM (n = 12), S-type (n = 8), X-type (n = 3), Haarlem (n = 2), and EAI (n = 1). A further nine strains were found and appear to form a previously undefined branch that was denominated Kiribati-H37Rv-like. Two of 73 (3%) strains, both belonging to the Euro-American super lineage, could not be further assigned. Overall, SNP typing confirmed that 36 of 73 (49%) strains belong to the Euro-American super lineage (all except Beijing and EAI strains, data not shown). The phylogenetic tree (Figure 1) describes the diverse lineages observed in Kiribati. These were confirmed using an MST (Figure 2) that identified all lineages as clonal complexes.

**Figure 3:**
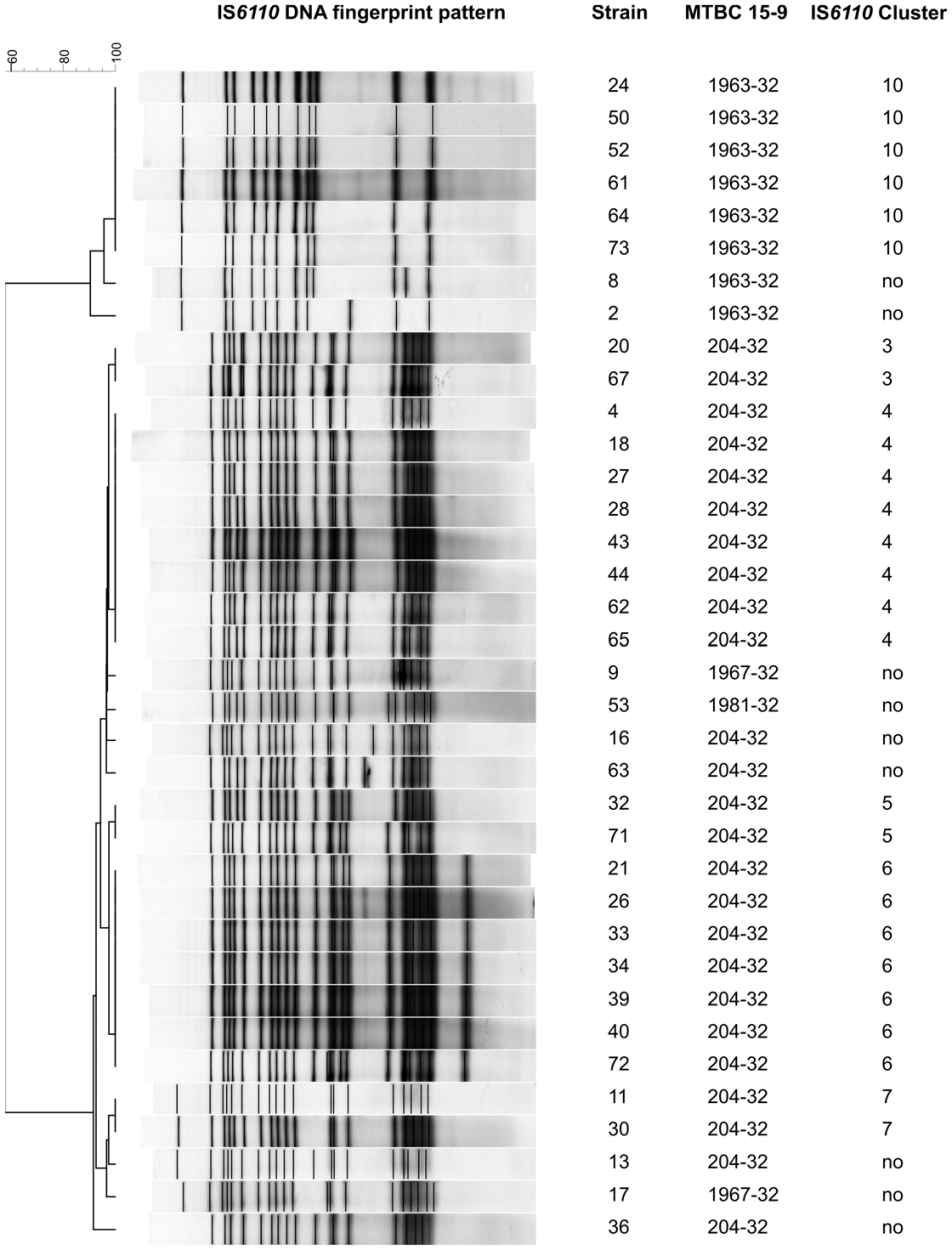
IS*6110* clustering patterns, DNA fingerprinting pattern and MTBC 15-9 classification of Kiribati isolated Beijing strains. The classification of Beijing strains in two major branches is further supported by completely different IS*6110* fingerprint patterns showing a higher copy number for Beijing-B isolates (>17 bands) compared Beijing-A isolates (<13 bands).

Thirty-six (49%) of the 73 Kiribati MTBC strains belonged to the Beijing lineage (confirmed by the absence of particular regions of difference, RD105 and RD207, data not shown). The absence of RD 207 is responsible for the typical deletion of spacer 1–34 in the DR locus of MTBC and in combination with the RD105 deletion characteristic for all Beijing family strains [18]. Interestingly, the Beijing strains circulating in Kiribati were clearly separated in two branches based on 24 loci MIRU-VNTR data (group Beijing-A and -B; Figure 1). Analysis of RD150 and RD181 revealed that strains of the more successful branch B (n = 28; all four RDs deleted) are more recently evolved compared to strains of branch A that are more ancestral (n = 8, only RD105 and RD207 deleted). The successive deletion of RD181 and RD150 subdivide the Beijing family and was shown to be a robust marker reflecting the phylogeny of Beijing family strains [18]. The classification of Beijing strains in two major branches is further supported by completely different IS*6110* fingerprint patterns showing a higher copy number for Beijing-B isolates (>17 bands) compared Beijing-A isolates (<13 bands) (Figure 3).

Next a cluster-analysis was performed to identify strains with genotyping profiles matching 100% with at least one other strain (100% identical spoligotyping, MIRU-VNTR-typing patterns, and/or IS*6110* DNA fingerprint pattern). Isolates were defined as non-clustered if they had unique, non-shared genotyping results. When only MIRU-VNTR typing and spoligotyping data were considered, 55 (75%) of the 73 analysed strains were grouped in 8 different clusters, ranging in size from 2 to 26 strains (Figure 1). However, when IS*6110* genotyping data were also considered, only 43 (59%) of 73 strains were clustered, each involving 2 to 7 isolates (Table S1). This reflects the much higher discriminatory power of IS*6110* typing in Beijing strains, that resulted in the 25 strains previously considered as a MIRU cluster (MIRU cluster 6, MTBC 15-9 type 204-32) as being split into 5 smaller clusters and 4 orphan strains by small differences in their IS*6110* patterns (Figure 3).

The finding that still 59% of Kiribati MTBC strains were involved in clusters suggests a high rate of ongoing recent transmission in the study area. We therefore looked for associations between patient demographics and cluster classification. There was a tendency for patients infected with a clustered MTBC strain to live in a more crowded home (median: 5 people living per room, interquartile range (IQR): 1.6–15) compared with those infected with a non-clustered isolate (median: 3.1 people per room, IQR: 0.8–14; p = 0.1, Wilcoxon rank-sum test). However, the factor most strongly associated with clustering was having TB caused by modern and more ancestral Beijing strains. Twenty-seven (62.8%) of 43 clustered strains were of Beijing lineage, compared with 9 (30%) of 30 non-clustered isolates (p = 0.006, Chi square test). On logistic regression modelling, having a Beijing strain was the only factor independently associated with being in a cluster of TB cases (Odds Ratio 3.9, 95% confidence intervals 1.5–10.7, p = 0.005).

## Discussion

This is the first molecular epidemiologic study of MTBC strains from Kiribati, a Western Pacific island nation experiencing very high rates of pulmonary tuberculosis. Data regarding patterns of MTBC transmission are urgently needed to guide effective local control measures. We show that strains from multiple MTBC lineages are circulating in Kiribati, with the most prevalent being strains of Beijing lineage and that one novel phylogenetic lineage has emerged (denoted Kiribati-H37RV-like). Strains from the Beijing lineage are widespread geographically and occur more frequently in clusters, suggesting enhanced transmission. Importantly, we found little evidence of resistance to first line anti-tuberculous agents in Kiribati MTBC strains.

The MTBC population present in Kiribati is composed almost entirely of an equal proportion of strains of the Beijing and Euro-American lineages. Interestingly, the Beijing strains are divided into two distinct branches, namely, branch-A and -B. Beijing-B strains, exhibiting the deletions RD105, RD207, RD181 and RD150 accounted for the majority of the Kiribati Beijing strains observed and were associated with clustering (likely recent transmission). This was not the case for the more ancestral Beijing-A strains with RD105 and RD207 deletions but intact RD181 and RD150. When we compared the IS*6110* profile of the Beijing branches, all Beijing-B strains have a high band number profile as usually found for W-Beijing strains from Eastern Europe and Asia [19] while Beijing-A strains have a more unusual IS*6110* pattern with a low band number (Figure 3).

The subdivision of Beijing strains in different branches is an interesting finding that is in accordance with previous studies [18], [20], [21]. Our data further support the hypothesis that more recently evolved branches of the Beijing lineage are more adapted to spread and cause disease than more ancestral branches [20], [22]. However, further studies investing larges sets of Beijing strains with valid phylogenetic markers are needed to further proof this and define pathobiological differences between different branches of the Beijing lineage e.g. with respect to transmission.

The Euro-American strains branch into previously described lineages such as LAM, X-type and Haarlem as well as the newly described lineage Kiribati_H37Rv_like strain reported here. Several other groups have also found region specific sub-lineages among Euro-American strains [23]–[25]. The very wide global distribution of Euro-American strains was not fully recognized until recently, largely because their valid identification is not possible by spoligotyping and IS*6110* typing alone, but requires 24-loci MIRU-VNTR typing, ideally in combination with SNP typing as performed here [10]. More comprehensive investigations of the global and regional population structure of the Euro-American super-linage are needed for valid descriptions of the different sub-lineages and associations with clinical characteristics such as transmissibility or drug resistance, as already found for strains of the Beijing lineage in various settings [24].

In MTBC epidemiology, MIRU-VNTR and spoligotyping are increasingly being used as first line methods for their ease, discriminatory power, and digital analysis of generated data [10], [26], [27] compared with the gold standard IS*6110* typing method. We found a reduced discriminatory power of 24-loci MIRU-VNTR compared to IS*6110* typing especially in Beijing lineages. Beijing strains of the clonal complex with MIRU 15-9 type 204-32 were only discriminated using IS*6110*, revealing five different RFLP clusters and four orphan strains. This finding confirms previous observations by us and others that the discriminatory power of 24-loci-MIRU-VNTR typing is less than that of IS*6110* typing for strains of particular groups of the Beijing lineage such as 204-32 or 94-32 [20], [24]. In these cases, 24-loci MIRU-VNTR typing is inadequate for accurately identifying transmission chains of Beijing strains, and thus needs to be complemented either by IS*6110* typing or combinations of MIRU-VNTR loci more variable in Beijing strains. However, it should be noted that the same phenomenon can be seen vice versa, meaning that clusters with strains having low IS*6110* band numbers (less than four bands) e.g. *M. bovis* or *M. tuberculosis* EAI are split by 24-loci-MIRU-VNTR typing [13], [25].

Beijing strains appear to be endemic in Kiribati; the high cluster rate and the overall close relationship strongly suggest ongoing endemic spread. The majority of clusters are formed by strains of the Beijing lineage (27 out of 43 clustered isolates), followed by one larger cluster formed by 6 S-type strains. Accordingly, infection with a Beijing strain (especially Beijing-B) was found to be the strongest risk factor for being in a transmission chain. As patients infected with Beijing strains did not differ from patients with non-Beijing strain infection with regard to other patient characteristics, our data indicate a higher capability of Beijing-B strains to spread in this population and identifies them as one clear driver of the local TB epidemic. Furthermore, when cluster data were compared to patient geographic origin, there was no clear association (data not shown).

We observed very little evidence of resistance to first line anti-tuberculous agents in Kiribati, with only three strains demonstrating reduced susceptibility to STR. In all cases, patients had never previously been treated for tuberculosis, suggesting primary drug resistance. No strain was resistant to any other first line agent. This suggests an epidemic that has the potential to be controlled with active case finding and treatment with standard regimens. Our finding of many cases belonging to clusters indicates that active case finding and ongoing screening of close contacts, particularly where patients live in crowded conditions (one village on South Tarawa has a population density of 10,000 people/square km), may be required to break ongoing chains of transmission [7]. In the future, such efforts would ideally be aided by affordable rapid diagnostic tests, including systems (e.g. GeneXpert, Cepheid, Sunnyvale, CA) to monitor the emergence of resistance to first line drugs among MTBC strains circulating in Kiribati.

While this study provides an important snapshot of the molecular epidemiology of pulmonary tuberculosis in Kiribati, it has a number of limitations. In particular, although more than 90% of patients' sputum was smear positive for AFB (suggesting the vast majority of study participants had tuberculosis) fewer than half the sputa were culture positive for MTBC. A factor that may have influenced culture results in this study was transit time between specimen collection and its arrival at the diagnostic facility in Noumea. Flights out of Tarawa atoll only occur once per week, making transporting samples logistically challenging. Whilst prolonged transit times were associated with negative culture results, these were random events, not expected to influence the overall pattern of MTBC strains obtained and thus the MTBC strains cultured in this study are likely to be representative of those causing pulmonary TB in Kiribati. The study findings are also limited by the paucity of patients who were tested for HIV.

In conclusion, we report evidence of high rates of ongoing transmission of pulmonary TB in Kiribati, particularly among those infected with Beijing-B MTBC strains and in those living in crowded conditions. This provides the grounds for enhanced case finding amongst close contacts and continued supervised treatment of all identified cases using standard first-line drugs to reduce TB burden in Kiribati. The importance of the sub classification of the Beijing lineage in different branches needs to be further investigated.

## Supporting Information

Table S1 Table S1 containing MLVA MtbC 15-9 and IS*6110* RFLP cluster information and all VNTR profiles for the 73 strains investigated.
(XLS)
